# Left Vocal Cord Palsy Immediately After COVID-19 Despite No Tracheal Intubation

**DOI:** 10.7759/cureus.29766

**Published:** 2022-09-29

**Authors:** Ryo Kawaura, Masami Ohnishi

**Affiliations:** 1 Department of Head and Neck Surgery-Otolaryngology, Ogaki Municipal Hospital, Ogaki, JPN

**Keywords:** recurrent laryngeal nerve palsy, intubation, sars-cov-2, covid-19, unilateral vocal cord paresis

## Abstract

The cases of coronavirus disease 2019 (COVID-19) typically present with pulmonary and upper respiratory tract symptoms, but may also present with neurologic complications. Because severe cases are often intubated or ventilated, there are some reports of vocal cord palsy associated with intubation; however, there are few reports of recurrent nerve palsy without intubation management. We experienced a case of left vocal cord palsy following COVID-19 in a healthy young male patient with no previous medical history. The patient became aware of hoarseness symptoms three days after he was found to be COVID-19 positive, and an endoscopic examination of the larynx revealed left vocal cord palsy. Since the patient had no previous medical history and there were no lesions that could cause recurrent nerve palsy on neck-thorax imaging, it was considered likely that the patient had unilateral recurrent nerve palsy due to acute inflammation caused by severe acute respiratory syndrome coronavirus 2 (SARS-CoV-2) infection. Medication was started and his hoarseness became relieved. In vocal cord palsy occurring after COVID-19 illness, it is necessary to consider the presence of both vocal cord palsy related to tracheal intubation and recurrent nerve palsy associated with SARS-CoV-2 infection.

## Introduction

Severe acute respiratory syndrome coronavirus 2 (SARS-CoV-2) is a novel coronavirus discovered in Wuhan, China, at the end of 2019, resulting in a worldwide coronavirus disease 2019 (COVID-19) pandemic in 2020. SARS-CoV-2 adsorbs cells by binding the spike protein protruding from the viral particles to infection receptors on the host cell membrane, utilizing the angiotensin-converting enzyme-2 (ACE2) receptor. ACE2 receptors are known to be highly expressed in the lineage cells of the nasal epithelium [1]. Therefore, it is thought that the infection may spread from the nasal cavity and olfactory nerve to the brain stem. Other possible causes of neurologic complications include infection by hematogenous passage through the blood-brain barrier, encephalopathy due to immunologic abnormalities, vascular endothelial damage and thrombosis, and the involvement of abnormalities in the renin-angiotensin-aldosterone system [1]. Among peripheral neuropathies, facial nerve palsy and Guillain-Barre syndrome have been reported relatively frequently [2], but there are few reports of recurrent nerve palsy or vocal cord palsy alone. In addition, COVID-19 often requires tracheal intubation in severe cases, and there are even fewer reports of recurrent nerve palsy without intubation. 

## Case presentation

A 20-year-old male with no medical history by birth, not vaccinated against SARS-CoV-2, tested positive for COVID-19 after antigen testing by a medical practitioner. Three days later, he noticed hoarseness. Ten days later, he visited ENT clinic, where a laryngoscopy revealed paralysis of the left vocal cord.

**Figure 1:**
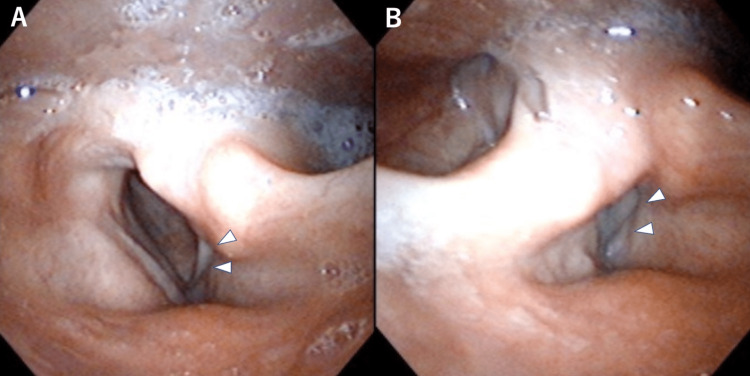
Endoscopic images of the left vocal cord palsy On inspiration, the right vocal cord opens enough, but the left vocal cord (white arrowhead) is fixed in paramedian position (A). On expiration, there is a gap between the bilateral vocal cords due to palsy of the left vocal cord (white arrowhead) (B).

The next day, he was referred to our hospital for further examination and treatment. During the conversation, a hoarse voice was heard. The endoscopy at the first visit to our hospital also showed left vocal cord palsy, and the left vocal cord was fixed in the paramedian position (Figure 1). No other significant lesions were found on endoscopy. Ultrasonography of the neck and CT of the chest revealed no lesion that could be the cause of recurrent nerve palsy. We considered the possibility of left vocal cord palsy associated with SARS-CoV-2 infection and decided to start oral mecobalamin treatment and follow-up.

Two weeks after his first visit to our hospital, his hoarseness gradually improved. Also one month after the patient's first visit, hoarseness in a conversation was almost reduced. The patient did not wish to be reevaluated by endoscopy or receive additional treatment. He has not received any SARS-CoV-2 vaccine after the COVID-19 illness yet.

## Discussion

COVID-19 is known to cause a variety of complications in addition to respiratory symptoms, neuropathy being one of them [3]. In Wuhan, 78 (36%) of 214 COVID-19 patients reported neurological complications such as headache, disorientation, sensory disturbance, and myopathy, with neurological complications being more likely to occur, especially in more severe cases [4]. In peripheral neuropathy, there are scattered reports of Guillain-Barré syndrome and Fisher syndrome [2].

In previous reports [5-6], vocal cord palsy has tended to increase in the elderly. The major causes are idiopathic (27.6-31.1%), tumor (15.4-31.1%), and surgery (28.9-39.7%). According to Iwata et al. [5], recurrent nerve palsy after endotracheal intubation occurring at sites unrelated to recurrent nerve or vagus nerve travel was found in 7.7% of all cases. It is thought that factors such as nerve compression due to cervical extension or flexion during intubation or intraoperatively, inappropriate choice of intubation tube, and excessive cuff pressure may have contributed to these cases. It has been indicated that a certain amount of idiopathic recurrent nerve palsy is caused by a viral infection; the prognosis for palsy in these cases is good, with previous reports showing recovery in 14 of 18 cases of unilateral paralysis and 10 of 11 cases of bilateral paralysis [5].

In addition to peripheral neuropathy, facial paralysis is often treated with oral vitamin B12. Also, regarding the topic of vitamin B12 deficiency, there have been reports of oral vitamin B12 treatment for vocal cord palsy [7-8]. Considering these points and the risks of other treatments, we began treatment with oral mecobalamin in this case.

There are limited reports [9-11] of vocal cord palsy occurring as a result of COVID-19 disease that does not involve intubation. In this case, vocal cord palsy occurred three days after the positive SARS-CoV-2 identification, earlier than in the literature reported previously. It has been suggested that vocal cord palsy after COVID-19 may be due to nerve infiltration of SARS-CoV-2 or inflammation associated with an immune response. Given that the time after COVID-19 onset can range from a few days to more than two weeks (Table 1), several mechanisms of palsy due to SARS-CoV-2 infection may exist. Furthermore, Rapoport et al. [12] published a case series of vocal cord palsy after COVID-19, in which 16 cases of unilateral or bilateral vocal cord paralysis were observed. Although the duration between COVID-19 and the onset of palsy was not specified in this case series, it suggests that the number of cases of vocal cord palsy after COVID-19 is higher than previously reported.

**Table 1:** Case reports of vocal cord palsy occurring as a result of COVID-19 without intubation

Reference No.	Author	Age	Sex	Side	Time after COVID onset	Medications	Progress
[9]	Korkmaz and Güven	57	female	left	15 days	Voice therapy injection into the left vocal cord	Improvement of voice
[10]	Tin et al.	58	male	left	43 days	Injection with Prolaryn Gel	Scheduled to be treated
[11]	Coggins et al.	18	female	right	up to 10 days	Observation	Recovered

Moreover, vocal cord palsy after COVID-19 vaccination as well as COVID-19 morbidity has also been reported [13-14]. However, the Vaccine Adverse Event Reporting System (VAERS) database indicates that vocal cord palsy has also been reported to occur with vaccination against influenza, shingles, pneumococcus, and hepatitis B [15-16]. Future studies are required to determine whether the COVID-19 vaccination is more likely to cause vocal cord palsy as compared to other vaccines.

## Conclusions

Vocal cord palsy may occur after COVID-19, even if intubation has not been performed. Although there have been only a few reports so far, there may be actually a certain number of cases in which paralysis is missed even though hoarseness symptoms are present. As in cases of paresis caused by other viral infections, the prognosis is often good, although some cases may require therapeutic intervention such as injections into the vocal cords, so further reports are needed.
